# Supplementary material for: Inflammatory diseases and risk of lung cancer among individuals who have never smoked
Source: Nat Commun. 2025 Jun 2;16:5095. doi: 10.1038/s41467-025-56803-z (PMC12130270; doi:10.1038/s41467-025-56803-z)
Supplement: Supplementary file 1 — Supplementary Information [file 41467_2025_56803_MOESM1_ESM.pdf]

Supplementary information for “Inflammatory diseases and risk of lung cancer among individuals who have never smoked”

Supplementary Methods: Hierarchical analyses ... 2

Supplementary Figure 1: Forest plots showing the associations between the medical conditions identified in the 1-10 years (left) and 10-32 years (right) prior to selection and lung cancer in individuals who have never smoked. Data are presented as adjusted odds ratios with 95% confidence intervals from conditional logistic regression models. Estimates in blue are the associations from the discovery (CPRD-GOLD) dataset (1-10 years: 1,581 LCINS cases and 14,318 never smoking controls, 10-32 years: 1,008 LCINS cases and 9,093 never smoking controls) and those in red are the associations in the validation dataset (CPRD-Aurum) (1-10 years: 2,188 LCINS cases and 19,597 never smoking controls, 10-32 years: 1,455 LCINS cases and 12,931 never smoking controls). Source data are provided as a Source Data File. ... 3

Supplementary Table S1: Adjusted odds ratios (aORs) and 95% confidence intervals (CIs) estimated from conditional logistic regression models for the association between disease categories and subcategories and LCINS in the discovery dataset (CPRD-GOLD). Multiple testing corrections were applied for conditions diagnosed 1-10 years prior to lung cancer in non-smokers (LCINS). No corrections were conducted for conditions diagnosed 10-32 years prior to LCINS, as these were secondary analyses. ... 4

Supplementary Table S2: Associations between medical conditions and LCINS in the discovery (CPRD-GOLD) dataset 1-10 years before selection adjusted for body mass index socioeconomic status (index of multiple deprivation) ... 6

Supplementary Table S3: Examining potential heterogeneity of gastritis and anemia based on Aurum codes ... 7

Supplementary Table S4: Statistical power calculations accounting for multiple testing and varying assumptions. The table illustrates that the study power depends on both the number and prevalence of conditions and medications within the population. Based on our preliminary estimates, we will have 80% power to detect odds ratios from approximately <1.27 to 2.30, accounting for multiple comparisons, depending on the prevalence of each factor in the case group. ... 8

Supplementary Table S5: Hierarchy of primary conditions and subcategories for the discovery stage ... 9

Supplementary Methods: Hierarchical analyses

For each primary category of conditions, we fit two-stage hierarchical logistic regression models to the conditions (subcategories) that it included (Witte et al, 1994). The model is formulated as follows. Letting Y=1 for a case and Y=0 for a non-case (control), the first stage of the logistic regression model has the form

$$p = P(Y = 1|X, W) = \frac{\exp(\alpha + \beta\mathbf{X} + \gamma\mathbf{W})}{1 + e\exp(\alpha + \beta\mathbf{X} + \gamma\mathbf{W})},$$

where **X** stands for the vector of individual subcategories in the primary category and **W** denotes a vector of other independent variables, i.e., sex, age, year of diagnosis. Each coefficient β_j in $\boldsymbol{\beta}$ represents the impact of the jth subcategory X_j on Y, and γ is the vector of log-odds ratios of the other covariates. In the second stage, we modeled each β_j using the linear model $\beta_j = \pi + \delta_j$, where π is the overall group mean. We assume that subcategories (conditions) within a group are exchangeable, and model the δ_j's as independent normal random variables with mean zero and variance τ. The δ_j's stand for residual effects that may arise from differences in the effects of conditions *within the same primary category.* We also present estimated individual conditions effects β_j by combining the fixed and predicted random effects for the individual conditions. In the hierarchical model, all variables are fit simultaneously, and therefore, each estimated coefficient is adjusted for all other conditions present in the model. Thus, no multiple testing adjustment is needed for the p-values. The models were fitted using PROC GLIMMIX (SAS 9.1).

Supplementary Figure 1: Forest plots showing the associations between the medical conditions identified in the 1-10 years (left) and 10-32 years (right) prior to selection and lung cancer in individuals who have never smoked. Data are presented as adjusted odds ratios with 95% confidence intervals from conditional logistic regression models. Estimates in blue are the associations from the discovery (CPRD-GOLD) dataset (1-10 years: 1,581 LCINS cases and 14,318 never smoking controls, 10-32 years: 1,008 LCINS cases and 9,093 never smoking controls) and those in red are the associations in the validation dataset (CPRD-Aurum) (1-10 years: 2,188 LCINS cases and 19,597 never smoking controls, 10-32 years: 1,455 LCINS cases and 12,931 never smoking controls). Source data are provided as a Source Data File.

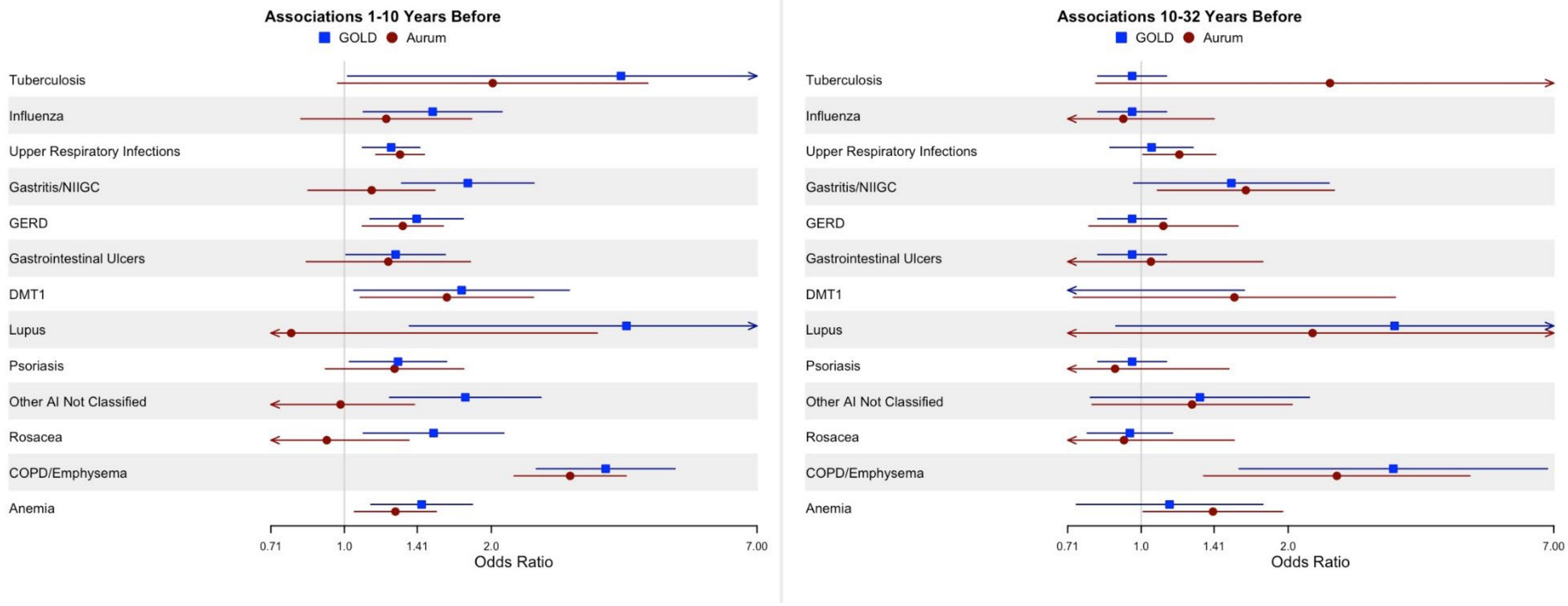

Supplementary Table S1: Adjusted odds ratios (aORs) and 95% confidence intervals (CIs) estimated from conditional logistic regression models for the association between disease categories and subcategories and LCINS in the discovery dataset (CPRD-GOLD). Multiple testing corrections were applied for conditions diagnosed 1-10 years prior to lung cancer in non-smokers (LCINS). No corrections were conducted for conditions diagnosed 10-32 years prior to LCINS, as these were secondary analyses.

DISEASES	DISCOVERY* (1-10 YEARS PRIOR TO SELECTION)				DISCOVERY* (10-32 YEARS PRIOR TO SELECTION)			
	No. (%) in cases	No. (%) in controls	aOR (95% CI)	p	No. (%) in cases	No. (%) in controls	aOR (95% CI)	p
**1. Infections and Inflammation**	1022 (64.6)	8703 (60.8)	1.26 (1.12,1.42)	1.41E-04	405 (40.2)	3701 (40.7)	1.06 (0.90,1.25)	0.4651
Pneumonia	23 (1.5)	153 (1.1)	1.38 (0.88,2.16)	0.155	8 (0.8)	43 (0.5)	1.79 (0.83,3.85)	0.1369
Affecting the heart	0 (0.0)	8 (0.1)	-	-	0 (0.0)	5 (0.1)	-	-
Affecting the head and neck	366 (23.1)	3516 (24.6)	0.94 (0.83,1.07)	0.371	177 (17.6)	1594 (17.5)	1.06 (0.87,1.29)	0.5722
Meningitis	0 (0.0)	6 (0.0)	-	-	NR	5 (0.1)	1.58 (0.18,13.60)	0.675
Hepatitis	NR	7 (0.0)	1.58 (0.19,13.19)	0.673	0 (0.0)	NR	-	-
Tuberculosis	NR	6 (0.0)	3.89 (0.96,15.81)	0.057	NR	18 (0.2)	1.34 (0.30,5.90)	0.7012
Herpes	7 (0.4)	68 (0.5)	0.96 (0.44,2.10)	0.917	9 (0.9)	36 (0.4)	2.51 (1.18,5.32)	0.0168
Affecting urinary tract	220 (13.9)	1962 (13.7)	1.02 (0.87,1.19)	0.819	86 (8.5)	792 (8.7)	1.03 (0.80,1.32)	0.8412
Affecting female sex	17 (1.1)	144 (1.0)	1.09 (0.65,1.82)	0.741	8 (0.8)	98 (1.1)	0.79 (0.38,1.64)	0.5216
Affecting limbs	63 (4.0)	581 (4.1)	0.96 (0.73,1.26)	0.786	22 (2.2)	181 (2.0)	1.21 (0.76,1.91)	0.4209
Affecting Skin	199 (12.6)	1639 (11.4)	1.15 (0.98,1.35)	0.098	68 (6.7)	548 (6.0)	1.16 (0.88,1.53)	0.2813
Affecting Bone	5 (0.3)	65 (0.5)	0.68 (0.27,1.71)	0.416	NR	23 (0.3)	1.60 (0.55,4.66)	0.3875
Septicemia	NR	23 (0.2)	0.68 (0.16,2.89)	0.599	NR	6 (0.1)	1.24 (0.15,10.41)	0.8416
Encephalitis	0 (0.0)	0 (0.0)	-	-	NR	NR	8.18 (0.51,130.79)	0.1374
Influenza	34 (2.2)	198 (1.4)	1.57 (1.07,2.31)	0.022	24 (2.4)	175 (1.9)	1.35 (0.85,2.13)	0.2045
Malaria	11 (0.7)	111 (0.8)	0.85 (0.44,1.65)	0.633	7 (0.7)	27 (0.3)	2.32 (0.94,5.73)	0.0683
Herpes Zoster/Shingles	60 (3.8)	564 (3.9)	0.96 (0.73,1.26)	0.755	31 (3.1)	245 (2.7)	1.24 (0.84,1.84)	0.2754
Dental problems	8 (0.5)	60 (0.4)	1.24 (0.59,2.60)	0.576	NR	18 (0.2)	0.49 (0.07,3.68)	0.4872
Non-upper respiratory infections	218 (13.8)	1734 (12.1)	1.21 (1.03,1.42)	0.019	69 (6.8)	505 (5.6)	1.35 (1.02,1.79)	0.0337
Upper respiratory infections	325 (20.6)	2599 (18.2)	1.18 (1.03,1.35)	0.021	135 (13.4)	1082 (11.9)	1.23 (0.99,1.53)	0.0608
Arthritis	36 (2.3)	330 (2.3)	0.99 (0.70,1.41)	0.969	18 (1.8)	112 (1.2)	1.59 (0.94,2.69)	0.0846
Gout	47 (3.0)	442 (3.1)	0.94 (0.69,1.29)	0.715	18 (1.8)	118 (1.3)	1.49 (0.89,2.49)	0.1274
Autoimmune conditions*	222 (14.0)	1674 (11.7)	1.28 (1.10,1.49)	0.002	60 (6.0)	626 (6.9)	0.89 (0.68,1.18)	0.4317
Inflammatory Bowel Disease	8 (0.5)	51 (0.4)	1.47 (0.69,3.10)	0.317	NR	29 (0.3)	0.69 (0.16,2.91)	0.6148
Rheumatoid arthritis	23 (1.5)	191 (1.3)	1.08 (0.70,1.68)	0.728	7 (0.7)	80 (0.9)	0.83 (0.38,1.82)	0.6413
Psoriasis	30 (1.9)	205 (1.4)	1.35 (0.91,2.00)	0.135	8 (0.8)	95 (1.0)	0.75 (0.36,1.55)	0.4317
Hypothyroidism	78 (4.9)	726 (5.1)	1.02 (0.80,1.30)	0.889	23 (2.3)	271 (3.0)	0.79 (0.51,1.22)	0.2908
Diabetes Mellitus Type 1	14 (0.9)	69 (0.5)	1.78 (0.99,3.19)	0.052	NR	30 (0.3)	0.29 (0.04,2.12)	0.2218
Lupus	5 (0.3)	11 (0.1)	4.54 (1.55,13.29)	0.006	NR	6 (0.1)	5.44 (1.29,22.86)	0.0209
Idiopathic Thrombocytopenic Purpura	NR	NR	5.46 (0.91,32.80)	0.064	NR	NR	4.78 (0.43,53.05)	0.2031
Other autoimmunne conditions NOS	35 (2.2)	190 (1.3)	1.67 (1.16,2.41)	0.006	13 (1.3)	73 (0.8)	1.70 (0.93,3.10)	0.0841
Alopecia	6 (0.4)	49 (0.3)	1.19 (0.50,2.81)	0.691	NR	17 (0.2)	1.99 (0.57,6.95)	0.2792
Polymylagia rheumatica	24 (1.5)	194 (1.4)	1.14 (0.74,1.75)	0.561	5 (0.5)	56 (0.6)	0.94 (0.37,2.39)	0.9024
Rosacea	33 (2.1)	210 (1.5)	1.44 (0.99,2.10)	0.06	6 (0.6)	90 (1.0)	0.63 (0.28,1.46)	0.2818
Related to autoimmune conditions	5 (0.3)	39 (0.3)	1.27 (0.50,3.23)	0.619	NR	10 (0.1)	1.75 (0.38,8.18)	0.4744
Gastrointestinal disease (GI)*	360 (22.8)	2821 (19.7)	1.25 (1.10,1.42)	0.001	132 (13.1)	1157 (12.7)	1.08 (0.88,1.32)	0.4483
Diverticulitis	66 (4.2)	547 (3.8)	1.13 (0.87,1.48)	0.356	17 (1.7)	176 (1.9)	0.86 (0.52,1.43)	0.5665
Haemmroids	74 (4.7)	626 (4.4)	1.10 (0.86,1.41)	0.456	32 (3.2)	313 (3.4)	0.97 (0.66,1.41)	0.8578
Dyspepsia	97 (6.1)	812 (5.7)	1.09 (0.88,1.37)	0.426	31 (3.1)	299 (3.3)	1.01 (0.69,1.49)	0.9529
Ulcer	20 (1.3)	116 (0.8)	1.53 (0.94,2.48)	0.088	7 (0.7)	71 (0.8)	0.93 (0.42,2.03)	0.8491
Miscellaaneous GI	36 (2.3)	276 (1.9)	1.23 (0.86,1.75)	0.254	15 (1.5)	110 (1.2)	1.33 (0.77,2.31)	0.3063
Oseophagitis	26 (1.6)	171 (1.2)	1.32 (0.87,2.01)	0.196	16 (1.6)	94 (1.0)	1.60 (0.93,2.74)	0.0905
Infectious origin	20 (1.3)	145 (1.0)	1.26 (0.78,2.02)	0.345	8 (0.8)	93 (1.0)	0.80 (0.38,1.67)	0.5558
Cholecystitis	NR	17 (0.1)	0.58 (0.08,4.43)	0.597	0 (0.0)	NR	-	-
Acute cholecystitis	9 (0.6)	57 (0.4)	1.57 (0.77,3.20)	0.218	2 (0.2)	19 (0.2)	1.01 (0.23,4.38)	0.9913
Gastro-oesophageal reflux disease	94 (5.9)	663 (4.6)	1.39 (1.11,1.74)	0.004	23 (2.3)	193 (2.1)	1.09 (0.70,1.71)	0.6941
Helicobacter infection	11 (0.7)	121 (0.8)	0.81 (0.44,1.51)	0.51	9 (0.9)	54 (0.6)	1.52 (0.74,3.10)	0.2533
Adenitis	NR	NR	4.96 (0.81,30.46)	0.084	NR	NR	5.47 (0.50,60.40)	0.1658
Calculus	NR	NR	6.22 (0.84,45.78)	0.073	0 (0.0)	NR	-	-
Gastritis & Non-infective gastroenteritis and colitis	50 (3.2)	284 (2.0)	1.64 (1.20,2.23)	0.002	21 (2.1)	109 (1.2)	1.81 (1.12,2.92)	0.0154
GI - not classified	5 (0.3)	32 (0.2)	1.65 (0.63,4.30)	0.305	NR	11 (0.1)	1.82 (0.39,8.51)	0.4461
Chronic Obstructive Pulmonary Disease/Emphysema	56 (3.5)	176 (1.2)	2.91 (2.12,3.99)	0	11 (1.1)	24 (0.3)	4.54 (2.18,9.46)	5.52E-05
Other Infections or inflammatory disease NOS	16 (1.0)	102 (0.7)	1.37 (0.80,2.34)	0.251	5 (0.5)	51 (0.6)	0.88 (0.35,2.21)	0.7804
**2. Cardiovascular Disease (CVD)**	890 (56.3)	7717 (53.9)	1.14 (1.01,1.28)	0.03	347 (34.4)	3212 (35.3)	1.02 (0.86,1.22)	0.792
Valve Problems	13 (0.8)	107 (0.7)	1.11 (0.62,2.01)	0.722	NR	9 (0.1)	3.33 (0.88,12.64)	0.076
Coronary Heart Disease (CHD)	215 (13.6)	1372 (9.6)	1.53 (1.30,1.80)	3.00E-07	47 (4.7)	399 (4.4)	1.12 (0.81,1.54)	0.496
CHD-Angina	91 (5.8)	562 (3.9)	1.46 (1.15,1.85)	0.002	21 (2.1)	235 (2.6)	0.83 (0.52,1.31)	0.413
CHD-Infarction	107 (6.8)	645 (4.5)	1.51 (1.22,1.88)	2.08E-04	32 (3.2)	263 (2.9)	1.19 (0.81,1.75)	0.366

CHD-Other	54 (3.4)	399 (2.8)	1.35 (1.00,1.83)	0.051	5 (0.5)	41 (0.5)	1.10 (0.43,2.82)	0.844
Heart Failure (HF)	60 (3.8)	363 (2.5)	1.50 (1.13,2.00)	0.006	10 (1.0)	65 (0.7)	1.42 (0.72,2.80)	0.315
HF-Precursor to heart failure	10 (0.6)	98 (0.7)	0.95 (0.49,1.83)	0.872	NR	20 (0.2)	1.92 (0.65,5.72)	0.241
HF-Heart Failure	51 (3.2)	279 (1.9)	1.63 (1.19,2.23)	0.002	7 (0.7)	47 (0.5)	1.34 (0.60,3.02)	0.476
Hypertension	488 (30.9)	4519 (31.6)	0.98 (0.87,1.11)	0.794	166 (16.5)	1511 (16.6)	1.03 (0.86,1.25)	0.731
Arrythmia	114 (7.2)	889 (6.2)	1.20 (0.98,1.48)	0.085	24 (2.4)	173 (1.9)	1.29 (0.83,2.00)	0.259
Dyslipidemia	425 (26.9)	3834 (26.8)	1.02 (0.88,1.18)	0.787	207 (20.5)	2006 (22.1)	0.94 (0.75,1.18)	0.573
Peripheral Vascular Disease	20 (1.3)	63 (0.4)	2.93 (1.74,4.96)	5.96E-05	NR	16 (0.2)	2.94 (1.03,8.38)	0.044
Other CVD	14 (0.9)	96 (0.7)	1.24 (0.70,2.19)	0.466	6 (0.6)	47 (0.5)	1.28 (0.53,3.10)	0.591
**3. Kidney Disease**	123 (7.8)	1094 (7.6)	1.07 (0.87,1.33)	0.518	11 (1.1)	55 (0.6)	2.67 (1.22,5.87)	0.014
**4. Thrombosis**	46 (2.9)	371 (2.6)	1.20 (0.87,1.64)	0.261	27 (2.7)	119 (1.3)	2.28 (1.47,3.52)	2.09E-04
Thrombosis (vein)	44 (2.8)	370 (2.6)	1.15 (0.83,1.58)	0.401	27 (2.7)	118 (1.3)	2.28 (1.47,3.52)	2.09E-04
**5. Allergic conditions**	337 (21.3)	2897 (20.2)	1.08 (0.95,1.23)	0.268	135 (13.4)	1216 (13.4)	1.06 (0.87,1.31)	0.551
Asthma	122 (7.7)	1011 (7.1)	1.14 (0.93,1.38)	0.212	47 (4.7)	443 (4.9)	1.02 (0.74,1.39)	0.916
Eczema	146 (9.2)	1266 (8.8)	1.05 (0.88,1.26)	0.586	52 (5.2)	437 (4.8)	1.16 (0.85,1.58)	0.34
Allergic Rhinitis/Hay fever	44 (2.8)	465 (3.2)	0.86 (0.63,1.19)	0.359	34 (3.4)	240 (2.6)	1.39 (0.95,2.04)	0.091
Other allergic conditions	87 (5.5)	661 (4.6)	1.17 (0.92,1.48)	0.201	25 (2.5)	289 (3.2)	0.80 (0.52,1.23)	0.304
**6. Diabetes Type 2/Type Unknown**	145 (9.2)	1237 (8.6)	1.09 (0.90,1.31)	0.386	35 (3.5)	284 (3.1)	1.12 (0.78,1.61)	0.542
Diabetes Type 2	86 (5.4)	836 (5.8)	0.94 (0.74,1.18)	0.592	20 (2.0)	207 (2.3)	0.87 (0.54,1.39)	0.551
**7. Polyps, Cysts, Fibrosis**	177 (11.2)	1424 (9.9)	1.17 (0.99,1.39)	0.07	86 (8.5)	650 (7.1)	1.26 (0.98,1.61)	0.071
Benign Neoplasms	63 (4.0)	504 (3.5)	1.20 (0.91,1.57)	0.19	31 (3.1)	194 (2.1)	1.51 (1.02,2.24)	0.038
Colorectal neoplasms	22 (1.4)	164 (1.1)	1.27 (0.81,2.01)	0.295	7 (0.7)	38 (0.4)	1.71 (0.75,3.90)	0.201
Specific to female sex	26 (1.6)	186 (1.3)	1.33 (0.87,2.03)	0.196	22 (2.2)	170 (1.9)	1.16 (0.72,1.88)	0.542
Skin cysts	68 (4.3)	566 (4.0)	1.10 (0.85,1.43)	0.481	26 (2.6)	258 (2.8)	0.90 (0.59,1.38)	0.628
Other cysts	36 (2.3)	287 (2.0)	1.16 (0.81,1.65)	0.428	15 (1.5)	87 (1.0)	1.60 (0.91,2.80)	0.1
**8. Eye Conditions**	191 (12.1)	1691 (11.8)	1.04 (0.88,1.24)	0.623	48 (4.8)	332 (3.7)	1.39 (1.00,1.92)	0.048
Glaucoma	43 (2.7)	386 (2.7)	1.01 (0.73,1.40)	0.958	19 (1.9)	109 (1.2)	1.61 (0.97,2.67)	0.064
Macular degeneration	18 (1.1)	176 (1.2)	0.91 (0.55,1.49)	0.703	NR	17 (0.2)	2.08 (0.70,6.19)	0.189
Other eye conditions	27 (1.7)	199 (1.4)	1.25 (0.83,1.88)	0.294	6 (0.6)	51 (0.6)	1.18 (0.50,2.79)	0.701
Cataracts	137 (8.7)	1212 (8.5)	1.05 (0.86,1.28)	0.642	25 (2.5)	187 (2.1)	1.26 (0.81,1.95)	0.303
**9. Varicose Veins**	49 (3.1)	405 (2.8)	1.08 (0.80,1.47)	0.615	30 (3.0)	250 (2.7)	1.13 (0.77,1.67)	0.535
**10. Stroke Related**	81 (5.1)	691 (4.8)	1.08 (0.85,1.38)	0.531	13 (1.3)	146 (1.6)	0.83 (0.46,1.48)	0.526
Stroke	56 (3.5)	443 (3.1)	1.19 (0.89,1.60)	0.234	9 (0.9)	84 (0.9)	0.99 (0.49,2.00)	0.977
Transient Ischemic Attack	37 (2.3)	346 (2.4)	0.96 (0.68,1.37)	0.835	5 (0.5)	73 (0.8)	0.66 (0.26,1.66)	0.377
**11. Osteoarthritis**	252 (15.9)	2285 (16.0)	1.01 (0.87,1.17)	0.926	97 (9.6)	961 (10.6)	0.91 (0.72,1.15)	0.422
**12. Osteoporosis**	92 (5.8)	784 (5.5)	1.13 (0.90,1.43)	0.292	25 (2.5)	160 (1.8)	1.57 (1.01,2.46)	0.047
**13.Anemia**	87 (5.5)	559 (3.9)	1.44 (1.13,1.83)	0.003	24 (2.4)	190 (2.1)	1.14 (0.74,1.78)	0.552
**14. Thyroid Disease**	40 (2.5)	278 (1.9)	1.41 (1.00,2.00)	0.049	11 (1.1)	79 (0.9)	1.24 (0.64,2.39)	0.52
**15. In situ cancers**	17 (1.1)	117 (0.8)	1.41 (0.84,2.36)	0.197	NR	43 (0.5)	0.63 (0.19,2.06)	0.443
**16. Gallbladder related**	36 (2.3)	327 (2.3)	1.05 (0.74,1.49)	0.791	18 (1.8)	178 (2.0)	0.97 (0.59,1.59)	0.891
**17. Psychiatric**	140 (8.9)	1165 (8.1)	1.11 (0.91,1.34)	0.301	67 (6.6)	599 (6.6)	1.09 (0.83,1.42)	0.557
Depresssion	111 (7.0)	959 (6.7)	1.05 (0.85,1.29)	0.662	58 (5.8)	554 (6.1)	1.02 (0.76,1.35)	0.922
Anxiety	34 (2.2)	247 (1.7)	1.36 (0.94,1.97)	0.106	10 (1.0)	120 (1.3)	0.80 (0.42,1.54)	0.505
Panic Disorder	15 (0.9)	108 (0.8)	1.35 (0.78,2.33)	0.284	5 (0.5)	43 (0.5)	1.05 (0.41,2.67)	0.925
Other psychiatric	25 (1.6)	165 (1.2)	1.42 (0.92,2.18)	0.114	9 (0.9)	36 (0.4)	2.52 (1.17,5.46)	0.019
**18. Hernia**	134 (8.5)	961 (6.7)	1.29 (1.06,1.56)	0.011	50 (5.0)	431 (4.7)	1.09 (0.80,1.48)	0.595
**19. Neurological Disorders**	161 (10.2)	1374 (9.6)	1.09 (0.91,1.29)	0.363	58 (5.8)	453 (5.0)	1.23 (0.92,1.65)	0.162
Epilepsy	15 (0.9)	126 (0.9)	1.16 (0.67,2.00)	0.597	8 (0.8)	41 (0.5)	2.05 (0.94,4.48)	0.072
Pain/Strange sensations	95 (6.0)	875 (6.1)	1.00 (0.80,1.26)	0.972	36 (3.6)	306 (3.4)	1.12 (0.78,1.61)	0.524
Parkinson's Disease	11 (0.7)	111 (0.8)	0.82 (0.44,1.55)	0.549	0 (0.0)	12 (0.1)	-	-
Multiple Sclerosis	NR	14 (0.1)	2.48 (0.81,7.55)	0.111	NR	9 (0.1)	2.22 (0.48,10.31)	0.309
**20. Dementia/Alzheimers**	26 (1.6)	404 (2.8)	0.55 (0.36,0.84)	0.005	NR	14 (0.2)	0.70 (0.09,5.40)	0.73
**21. Conditions affecting only male sex**	63 (4.0)	600 (4.2)	0.92 (0.70,1.22)	0.57	11 (1.1)	161 (1.8)	0.63 (0.33,1.18)	0.15
Erectile dysfunction	31 (2.0)	280 (2.0)	1.03 (0.70,1.53)	0.876	NR	32 (0.4)	0.90 (0.27,3.00)	0.866
Benign prostate hyperplasia	36 (2.3)	346 (2.4)	0.88 (0.62,1.27)	0.5	9 (0.9)	132 (1.5)	0.62 (0.31,1.26)	0.188
**22. Keratosis**	107 (6.8)	935 (6.5)	1.08 (0.87,1.34)	0.487	25 (2.5)	217 (2.4)	1.09 (0.71,1.68)	0.696
**23. Irritable Bowel Syndrome**	31 (2.0)	321 (2.2)	0.91 (0.62,1.32)	0.603	32 (3.2)	208 (2.3)	1.48 (1.01,2.18)	0.047
**24. Coditions affectinng only female sex**	61 (3.9)	487 (3.4)	1.22 (0.92,1.62)	0.177	35 (3.5)	323 (3.6)	0.98 (0.67,1.44)	0.913

*(not included in Hierarchical modeling)

Abbreviations: aOR, adjusted odds ratio; CI, confidence interval; AI, autoimmune; GERD, Gastroesophageal reflux disease; DMT1, Type 1 Diabetes; COPD, Chronic Obstructive Pulmo-ry Disorder; NIIGC, *Non-infective* inflammatory gastroenteritis and colitis; NR: Not reportable because of CPRD cell size (<5) reporting requirements.

aOR: Conditional regression model [Controls were individually matched to cases on year of birth (+/- 2 years); sex; general practice or region (general practice first, then region if we could not identify a control within the same practice); and year of practice registration (+/- 2 year)] odds ratios are adjusted for age (linear term).

Supplementary Table S2: Associations between medical conditions and LCINS in the discovery (CPRD-GOLD) dataset 1-10 years before selection adjusted for body mass index socioeconomic status (index of multiple deprivation)

	No. (%) in cases	No. (%) in controls	aOR (95% CI)	aOR1 (95% CI)
Infections and Inflammation				
Tuberculosis	NR	NR	1.78 (0.19,16.40)	1.77 (0.19,16.53)
Influenza	16(2.32)	68(1.08)	2.20 (1.22,3.98)	2.18 (1.20,3.95)
Upper Respiratory Infections [b]	144(20.87)	1175(18.70)	1.16 (0.94,1.42)	1.16 (0.94,1.42)
Gastritis/NIGC	15(2.17)	93(1.48)	1.41 (0.80-2.48)	1.44 (0.82-2.53)
GERD	41(5.94)	306(4.87)	1.31 (0.93-1.84)	1.29 (0.92-1.82)
Gastrointestinal Ulcers [c]	8(1.16)	50(0.80)	1.49 (0.69-3.21)	1.48 (0.68-3.20)
DMT1	7(1.01)	30(0.48)	2.69 (1.14-6.36)	2.67 (1.13-6.33)
Lupus	NR	NR	3.17 (0.33-30.51)	3.36 (0.35-32.44)
Psoriasis	11(1.59)	100(1.59)	1.06 (0.56-2.01)	1.07 (0.57-2.03)
Other AI not classified [d]	12(1.74)	83(1.32)	1.37 (0.74-2.55)	1.37 (0.73-2.54)
Rosacea	14(2.03)	83(1.32)	1.47 (0.82-2.63)	1.46 (0.81-2.62)
COPD/Emphysema	26(3.77)	70(1.11)	3.61 (2.23-5.86)	3.60 (2.21-5.85)
Anemia	39(5.65)	249(3.96)	1.55 (1.08-2.24)	1.56 (1.08-2.24)

Abbreviations: aOR, adjusted odds ratio; CI, confidence interval; NR: Not reportable because of CPRD cell size (<5) reporting requirements; NIGC, Non-infective gastroenteritis and colitis; GERD, Gastroesophageal reflux disease; DMT1, Diabetes Mellitus Type 1; AI, autoimmune; COPD, Chronic Obstructive Pulmonary Disorder.

aOR: Conditional regression model [Controls were individually matched to cases on year of birth (+/- 2 years); sex; general practice or region (general practice first, then region if we could not identify a control within the same practice); and year of practice registration (+/- 2 year)] odds ratios are adjusted for age (linear term).

aOR1: Further adjusted for body mass index (underweight, normal, overweight, obese, missing) and index of multiple deprivation in declies.

[a]Includes 690 never smoking lung cancer cases and 6,285 never smoking controls with at least 1 year of registration within a CPRD-GOLD primary care clinic with linkage to the index of multiple deprivation.

[b]Consists of terms indicative of bronchitis, tracheitis or upper respiratory infections

[c]Consists of terms indicative of peptic, gastric or duodenal ulcers

[d]Other autoimmune conditions not classified

Supplementary Table S3: Examining potential heterogeneity of gastritis and anemia based on Aurum codes

Associations between subtypes of Gastritis and LCINS in Aurum

Gastritis Codes	Gastritis associations: 1-10 years before			Gastritis Code Categories: 10-32 years before		
	No. (%) in cases	No. (%) in controls	aOR (95% CI)	No. (%) in cases	No. (%) in controls	aOR (95% CI)
"Identical" Gastritis/NIGC codes to GOLD	50 (2.29)	392 (2.00)	1.14 (0.84,1.53)	27 (1.86)	148 (1.14)	1.64 (1.08-2.49)
Gastritis/NIGC identical codes + similar codes	58 (2.65)	429 (2.19)	1.21 (0.92,1.60)	29 (1.99)	169 (1.31)	1.54 (1.03-2.31)
Autoimmune gastritis codes	NR	7 (0.04)	1.37 (0.17,11.18)	NR	NR	2.09 (0.23-18.92)
Acute Gastritis codes	9 (0.41)	81 (0.41)	1.03 (0.52,2.07)	7 (0.48)	48 (0.37)	1.11 (0.25-4.83)
Chronic gastritis codes	7 (0.32)	34 (0.17)	1.89 (0.84,4.27)	NR	16 (0.12)	0.91 (0.21,3.93)
Only Gastritis codes	53 (2.42)	407 (2.08)	1.17 (0.87,1.56)	28 (1.92)	162 (1.25)	1.54 (1.02-2.32)
Gastric Colitis codes	6 (0.27)	36 (0.18)	1.46 (0.61,3.48)	NR	12 (0.09)	3.40 (1.05-10.94)

Associations between subtypes of Anemia and LCINS in Aurum

Anemia Codes	Anemia associations 1-10 years before			Anemia Associations 10-32 years before		
	No. (%) in cases	No. (%) in controls	aOR (95% CI)	No. (%) in cases	No. (%) in controls	aOR (95% CI)
"Identical" Anemia codes to GOLD	128 (5.85)	896 (4.57)	1.27 (1.05,1.54)	44 (3.02)	283 (2.19)	1.40 (1.01-1.95)
Additional Anemia codes	NR	25 (0.13)	1.10 (0.33,3.67)	NR	NR	3.13 (0.33-30.15)
Megaloblastic anemia codes	0 (0.00)	NR	-	0 (0.00)	NR	-
Iron related Anemia codes	67 (3.06)	453 (2.31)	1.31 (1.01,1.71)	26 (1.79)	156 (1.21)	1.49 (0.97-2.29)
B12 related Anemia codes	NR	20 (0.10)	1.76 (0.60,5.19)	2 (0.14)	7 (0.05)	2.39 (0.49-11.53)
Folate related anemia codes	NR	6 (0.03)	1.68 (0.19,14.63)	0 (0.00)	NR	-
Pernicious Anemia codes	12 (0.55)	97 (0.49)	1.07 (0.59,1.96)	NR	34 (0.26)	1.07 (0.38-3.04)
Other Anemia codes	6 (0.27)	47 (0.24)	1.08 (0.46,2.55)	0 (0.00)	NR	-

Abbreviations: aOR, adjusted odds ratio; CI, confidence interval; NIGC, Non-infective gastroenteritis and colitis; NR: Not reportable because of CPRD cell size (<5) reporting requirements.

Supplementary Table S4: Statistical power calculations accounting for multiple testing and varying assumptions. The table illustrates that the study power depends on both the number and prevalence of conditions and medications within the population. Based on our preliminary estimates, we will have 80% power to detect odds ratios from approximately <1.27 to 2.30, accounting for multiple comparisons, depending on the prevalence of each factor in the case group.

Number of Conditions to examine	Prevalence in cases	Alpha	Power	Minimum Detectable OR
50	1%	0.001	80%	2.3
50	5%	0.001	80%	1.59
50	10%	0.001	80%	1.4
50	20%	0.001	80%	1.3
100	1%	0.0005	80%	2.14
100	5%	0.0005	80%	1.49
100	10%	0.0005	80%	1.35
100	20%	0.0005	80%	1.27

Supplementary Table S5: Hierarchy of primary conditions and subcategories for the discovery stage

Hierarchy of primary conditions (N) and subconditions	Tab for codes in Supplemental DiscoveryGoldCodes.xlsx
**1. Infections and Inflammation**	InfectInflam
Pneumonia	InfectInflam_Pneum
Affecting the heart	InfectInflam_Heart
Affecting the head and neck	InfectInflam_HN
Meningitis	InfectInflam_Menin
Hepatitis	InfectInflam_Hep
Tuberculosis	InfectInflam_TB
Herpes	InfectInflam_Herpes
Affecting urinary tract	InfectInflam_UT
affecting female sex	InfectInflam_OBGYN
Affecting limbs	InfectInflam_Limb
Affecting Skin	InfectInflam_Skin
Affecting Bone	InfectInflam_Bone
Septicemia	InfectInflam_Septi
Encephalitis	InfectInflam_Enceph
Influenza	InfectInflam_Flu
Malaria	InfectInflam_Malaria
Shingles	InfectInflam_Shingles
Dental problems	InfectInflam_Dental
Non-upper respiratory infections	InfectInflam_Resp
Upper respiratory infections	InfectInflam_Uresp
Arthritis	InfectInflam_Arthrit
Gout	InfectInflam_Gout
Autoimmune conditions*	InfectInflam_AI
Inflammatory Bowel Disease	InfectInflam_AI_IBD
Rhuematoid arthritis	InfectInflam_AI_RA
Psoriasis	InfectInflam_AI_Psor
Hypothyroidism	InfectInflam_AI_Lthy
Diabetes Mellitus Type 1	InfectInflam_AI_DMT1
Lupus	InfectInflam_AI_Lupus
idiopathic thrombocytopenic purpura	InfectInflam_AI_IDP
Other autoimmune conditions not classified	InfectInflam_AI_oth2
Alopecia	InfectInflam_AI_Alo
Polymylagia rheumatica	InfectInflam_AI_PolyR
Rosacea	InfectInflam_AI_Rose
Related to autoimmune conditions	InfectInflam_AI_NoCAT
Gastrointestinal disease (GI)*	InfectInflam_GI
Diverticulitis	InfectInflam_GI_Diver
Haemmroids	InfectInflam_GI_Haem
Dyspepsia	InfectInflam_GI_Dys
Ulcer	InfectInflam_GI_Ulc
Miscellaneous GI	InfectInflam_GI_Other
Oseophagitis	InfectInflam_GI_Ose
Of Infectious origin	InfectInflam_GI_Inf
Cholecystitis	InfectInflam_GI_Chol
Acute cholecystitis	InfectInflam_GI_Ichol
Gastro-oesophageal reflux disease	InfectInflam_GI_GERD
Helicobacter infection	InfectInflam_GI_HP
Adenitis	(InfectInflam_GI_Aden
Calculus	InfectInflam_GI_Calc
Gastritis & Non-infective gastroenteritis and colitis	InfectInflam_GI_GastrNIIGC
GI - not classified	InfectInflam_GI_NoCAT
Chronic Obstructive Pulmonnaary Disease/Emphysema	InfectInflam_COPD
Other Infections or inflammatory disease NOS	InfectInflam_Other
**2. Cardiovascular Disease (CVD)**	CVD
Valve Problems	CVD_ValveProblem
Coronary Heart Disease (CHD)*	CVD_CHD
CHD-Angina	CVD_CHD_Angina
CHD-Infarction	CVD_CHD_Infarction
CHD-Other	CVD_CHD_Other
Heart Failure (HF)*	CVD_HF
HF-Precursor to heart failure	CVD_HF_Precursor
HF-Heart Failure	CVD_HF_Failure
Hypertension	CVD_Hypertension
Arrythmia	CVD_Arrhythmia

Dyslipidemia	CVD_Dyslipidemia
Peripheral Vascular Disease	CVD_PVD
Other CVD	CVD_Other
**3. Kidney Disease**	KidneyDisease
**4. Thrombosis**	Thrombosis
Thrombosis (vein)	Thrombosis_Vein
**5. Allergic conditions**	Allergy
Asthma	Allergy_Asthma
Eczema	Allergy_Eczema
Allergic Rhinitis/Hay fever	Allergy_Hayfever
Other allergic conditions	Allergy_Other
**6. Diabetes Type 2/Type Unknown**	Diabetes
Diabetes Type 2	Diabetes_DMT2
**7. Polyps, Cysts, Fibrosis**	PolCystFibro
Benign Neoplasms	PolCystFibro_Bneo
Colorectal neoplasms	PolCystFibro_BNeo_CR
Specific to female sex	PolCystFibro_Fem
Skin cysts	PolCystFibro_CystSkin
Other cysts	PolCystFibro_CystOth
**8. Eye Conditions**	EyeConditions
Glaucoma	EyeConditions_Glau
Macular degeneration	EyeConditions_MacDeg
Other eye conditions	EyeConditions_Other
Cataracts	EyeConditions_Cata
**9. Varicose Veins**	VaricoseVeins
**10. Stroke Related**	StrokeRelated
Stroke	StrokeRelated_Stroke
Transient Ischemic Attack	StrokeRelated_TIA
**11. Osteoarthritis**	Osteoarthritis
**12. Osteoporosis**	Osteoporosis
**13.Anemia**	Anemia
**14. Thyroid Disease**	ThyroidDisease
**15. In situ cancers**	InSitu
**16. Gallbladder related**	Gallbladder
**17. Psychiatric**	Psychiatric
Depresssion	Psychiatric_Depress
Anxiety	Psychiartic_Anxiety
Panic Disorder	Psychiatric_Panic
Other psychiatric	Psychiatric_Other
**18. Hernia**	Hernia
**19. Neurological Disorders**	NeuroDis
Epilepsy	NeuroDis_Epilepsy
Pain/Strange sensations	NeuroDis_PainSens
Parkinson's Disease	NeuroDis_Park
Multiple Sclerosis	NeuroDis_MS
**20. Dementia/Alzheimers**	DementiaAlzheimers
**21. Conditions affecting only male sex**	MaleConditions
Erectile dysfunction	MaleConditions_ED
Benign prostate hyperplasia	MaleConditions_BPH
**22. Keratosis**	Keratosis
**23. Irritable Bowel Syndrome**	IBS
**24. Coditions affecting only female sex**	FemaleConditions

*** Not included in Hierarchical models**

Reference

Witte JS, Greenland S, Haile RW, Bird CL. Hierarchical regression analysis applied to a study of multiple dietary exposures and breast cancer. Epidemiology. 1994 Nov;5(6):612-21. doi: 10.1097/00001648-199411000-00009. PMID: 7841243.
