# Supplementary material for: Inflammatory diseases and risk of lung cancer among individuals who have never smoked
Source: Nat Commun. 2025 Jun 2;16:5095. doi: 10.1038/s41467-025-56803-z (PMC12130270; doi:10.1038/s41467-025-56803-z)
Supplement: Supplementary file 4 — Source Data [file 41467_2025_56803_MOESM4_ESM.zip › Source data files/readme.docx]

‘Source data file for Figure 1.xlsx’

‘Source data file for Supplementary Figure 1.xlsx’

‘Source data file S1_ValidationAurumCodes.xlsx’: codes used to identify medical conditions in the CPRD-Aurum dataset.

‘Source data file S2_DiscoveryGOLDCodes.xlsx’: codes used to identify medical conditions in the CPRD-GOLD dataset.

‘Source data file S3_MedicationsGOLD.xlsx’: list of medications in the CPRD-GOLD dataset.

‘Source data file S4_MedicationsAurum.xlsx’: list of medications in the CPRD-Aurum dataset.
